# Supplementary material for: Evolved bacterial resistance against fluoropyrimidines can lower chemotherapy impact in the Caenorhabditis elegans host
Source: eLife. 2020 Nov 30;9:e59831. doi: 10.7554/eLife.59831 (PMC7725501; doi:10.7554/eLife.59831)
Supplement: Transparent reporting form [file elife-59831-transrepform.docx]

***eLife’s* transparent reporting form**

We encourage authors to provide detailed information *within their submission* to facilitate the interpretation and replication of experiments. Authors can upload supporting documentation to indicate the use of appropriate reporting guidelines for health-related research (see [EQUATOR Network](http://www.equator-network.org/%20)), life science research (see the [BioSharing Information Resource](https://biosharing.org/)), or the [ARRIVE guidelines](http://www.plosbiology.org/article/info:doi/10.1371/journal.pbio.1000412) for reporting work involving animal research. Where applicable, authors should refer to any relevant reporting standards documents in this form.

If you have any questions, please consult our Journal Policies and/or contact us: [editorial@elifesciences.org](mailto:editorial@elifesciences.org).

**Sample-size estimation**

- You should state whether an appropriate sample size was computed when the study was being designed
- You should state the statistical method of sample size computation and any required assumptions
- If no explicit power analysis was used, you should describe how you decided what sample (replicate) size (number) to use

Please outline where this information can be found within the submission (e.g., sections or figure legends), or explain why this information doesn’t apply to your submission:

The number of populations evolved was chosen based on experimental microbial evolution convention and feasibility (incubator capacity). Analysis on evolved populations were performed on all evolved cultures. Analysis on evolved resistant clones was done after selecting a single resistant clone from a set of 10 randomly chosen clones from each evolved culture. The sampling method is shown in figure 3A and is fully described in the materials and methods section.

**Replicates**

- You should report how often each experiment was performed
- You should include a definition of biological versus technical replication
- The data obtained should be provided and sufficient information should be provided to indicate the number of independent biological and/or technical replicates
- If you encountered any outliers, you should describe how these were handled
- Criteria for exclusion/inclusion of data should be clearly stated
- High-throughput sequence data should be uploaded before submission, with a private link for reviewers provided (these are available from both GEO and ArrayExpress)

Please outline where this information can be found within the submission (e.g., sections or figure legends), or explain why this information doesn’t apply to your submission:

All experiments were done with biological duplicates or triplicates. All information addressing experimental replication can be found in the Material and Methods section.

**Statistical reporting**

- Statistical analysis methods should be described and justified
- Raw data should be presented in figures whenever informative to do so (typically when N per group is less than 10)
- For each experiment, you should identify the statistical tests used, exact values of N, definitions of center, methods of multiple test correction, and dispersion and precision measures (e.g., mean, median, SD, SEM, confidence intervals; and, for the major substantive results, a measure of effect size (e.g., Pearson's r, Cohen's d)
- Report exact p-values wherever possible alongside the summary statistics and 95% confidence intervals. These should be reported for all key questions and not only when the p-value is less than 0.05.

Please outline where this information can be found within the submission (e.g., sections or figure legends), or explain why this information doesn’t apply to your submission:

Statistical analysis methods and reporting for experiments can be found in the Material and Methods section. Statistical analysis we performed to detect barcode enrichments in the deletion library and differentially expressed genes in evolved strains. These statistical tests were performed internally by the DESeq2 and DEBRA packages and were based on biological replicates. We used conservative cutoff values for false-discovery-rate adjusted p-values (<0.1, detailed in the material and methods section).

The full list of enriched categories and statistical summary of the E. coli knock out screen can be found in supplementary table 2.

Differential expression analysis results of RNA-seq data (log2 fold change and FDR adjusted p-value) can be found in supplementary table 6.

(For large datasets, or papers with a very large number of statistical tests, you may upload a single table file with tests, Ns, etc., with reference to sections in the manuscript.)

**Group allocation**

- Indicate how samples were allocated into experimental groups (in the case of clinical studies, please specify allocation to treatment method); if randomization was used, please also state if restricted randomization was applied
- Indicate if masking was used during group allocation, data collection and/or data analysis

Please outline where this information can be found within the submission (e.g., sections or figure legends), or explain why this information doesn’t apply to your submission:

Experimenter classifying the nematode phenotypes was kept blind to the bacteria that were used as a food source. This is addressed in Materials and Methods: Drug toxicity in C. elegans.

**Additional data files (“source data”)**

- We encourage you to upload relevant additional data files, such as numerical data that are represented as a graph in a figure, or as a summary table
- Where provided, these should be in the most useful format, and they can be uploaded as “Source data” files linked to a main figure or table
- Include model definition files including the full list of parameters used
- Include code used for data analysis (e.g., R, MatLab)
- Avoid stating that data files are “available upon request”

Please indicate the figures or tables for which source data files have been provided:

A full list of barcode frequencies and enrichment from the E. coli screens (Figure 2) can be found in supplementary table 1. Similarly, the list of enriched gene categories can be found in supplementary table 2.

Details of growth rate values for E. coli knock out strains from Figure 2E can be found in supplementary table 3.

IC50 calculated values for all ancestor and evolved strains across each drug-media environment and strain growth rate (Figure 1C and Figure 3) can be found in supplementary table 4.

A full summary of the mutations and validated loss-of-function resistance (Figure 5 A-D) are detailed in supplementary table 5.

Differential expression analysis of evolved E. coli RNA-seq data (Figure 5 E-G) can be found in supplementary table 6 containing log2 fold change and FDR adjusted p-value of genes. Similarly, the list of enriched gene categories can be found in supplementary table 7.

Supplementary table 8 denotes C. elegan phenotype scoring (Figure 4). This table is not directly referenced in the manuscript.
